# Spectroscopic Insights into Nanodiamond–Doxorubicin Interactions in Drug Delivery Systems for Potential Cancer Treatment: “What Is Essential Is Invisible to the Eye”

**DOI:** 10.3390/pharmaceutics18040438

**Published:** 2026-04-01

**Authors:** Danica Jović, Branislav Jović, Ivana Borišev, Višnja Bogdanović, Aleksandar Djordjevic

**Affiliations:** 1 Department of Chemistry, Biochemistry, and Environmental Protection, Faculty of Sciences, University of Novi Sad, Trg Dositeja Obradovića 3, 21000 Novi Sad, Serbia; branislav.jovic@dh.uns.ac.rs (B.J.); aleksandar.djordjevic@dh.uns.ac.rs (A.D.); 2 Oncology Institute of Vojvodina, Put doktora Goldmana 4, 21204 Sremska Kamenica, Serbia; visnja.bogdanovic@gmail.com

**Keywords:** non-covalent drug delivery, nanocarriers, nanodiamonds, doxororubicin, cancer cell lines, UV–VIS spectroscopy, DLS measurements

## Abstract

**Background/Objectives**: Non-covalent nanocarrier-based systems have become a promising platform as they offer a strategy to improve the efficacy-safety profile of doxorubicin (DOX) without altering its chemical structure. Praised for biocompatibility and rich surface chemistry, nanodiamonds (NDs) have launched as nanocarriers of choice for advanced cancer therapy. By investigating DOX-ND physicochemical interactions, this work advances the structural understanding of a non-covalent potential anticancer system, which has not been quantitatively experimentally explored so far. **Methods**: To our knowledge, this is among the first studies combining ultraviolet–visible (UV–VIS) spectroscopy with spectral deconvolution to reveal the redistribution of different DOX species in the presence of NDs. Centrifugation-assisted analysis enabled differentiation between hypothetical labile and stable ND/DOX fractions. Adsorption kinetics was studied, and dynamic light scattering (DLS) measured particle size and zeta potential. In vitro screening was performed in non-malignant fibroblasts (MRC-5) and malignant melanoma (HS294T), glioblastoma (U251), and breast cancer (MCF-7) cells to evaluate ND/DOX combinations. **Results**: Centrifugation analysis revealed heterogeneous ND-DOX binding. Kinetic experiments showed fast multi-stage adsorption kinetics, best described by a bi-exponential decay function and the Weber–Morris model. DLS suggested stable systems with a particle size within 10–80 nm, predominantly around 20 nm, and positive zeta potential. Comparative in vitro screening demonstrated differential cellular responses across cell types, highlighting the relevance of ND/DOX interactions. **Conclusions**: The findings contribute to elucidating ND-DOX interactions relevant for the design and optimization of drug delivery systems, emphasizing the importance of spectroscopic insights for the design of nanodiamond-based drug delivery systems.

## 1. Introduction

Doxorubicin (DOX) has become a landmark of cancer investigation; however, the advances made so far have not resolved some of the major drawbacks of this drug, particularly toxicity, especially cardiotoxicity. Non-covalent nanocarrier-based drug delivery systems have been developed with the aim to handle this challenge and to enhance drug efficacy while minimizing systemic toxicity. Some of the most dominant advantages of non-covalent formulations over the covalent ones are the preserved drug integrity, smoother release, and the overall nanoformulation simplicity [1]. The main bonding forces within these systems between nanocarrier and drug are electrostatic interactions, hydrogen bonding, and π-π stacking. Owing to its planar aromatic structure and amphoteric nature, doxorubicin has a pronounced tendency to engage in non-covalent interactions and represents one of the best model drugs for such systems. Among other carbon-based nanocarriers, such as fullerenol, graphene, and carbon nanotubes [1,2], nanodiamonds (ND) have also emerged as promising drug delivery platforms [3]. They have been praised for their biocompatibility, high surface area and versatile surface chemistry, in combination with their ability to form non-covalent formulations [4]. Although nanodiamonds have been widely explored as carriers for doxorubicin [5,6,7,8], a comprehensive, quantitative understanding of the mechanisms governing ND/DOX interactions has been less systematically addressed, and the physicochemical drivers of these phenomena remain incompletely elucidated.

The nanoformulation’s efficacy stems not only from the efficacy of a drug and potential protective/enhancing action of nanocarrier, but also from synergistic interplay—a phenomenon known to depend on the properties of the system as a whole rather than on the properties of its individual components [9]. The latter underlines the need for detailed study of interactions within new nanoformulations of drugs. Unlocking the potential of non-covalent nanodiamond-based drug delivery systems is the process which requires the comprehensive understanding of the molecular interactions governing drug binding, the kinetics of loading, and their impact on the stability of such formulations.

In this research, we combined the application of spectroscopy (UV–VIS), dynamic light scattering (DLS), kinetic modelling, and cell-based assays to link the interactions with nanoformulation behavior and performance. To extend the physicochemical insights toward biological relevance, in vitro experiments were performed on four human cell lines, including the non-malignant fibroblasts (MRC-5) cell line and the malignant melanoma (HS294T), breast cancer (MCF-7), and glioblastoma (U251) cell lines.

The focus of this study was not on developing or improving a novel nanodrug delivery system, but rather on unveiling the fine interactions between nanodiamonds and doxorubicin, and examining how these interactions direct formulation stability and biological response. Insights such as this are essential for the development of efficient and safe nanodiamond-based drug delivery systems.

The novelty of our study is in the combination of complementary experimental and kinetic approach, combined with the spectra analyses including spectral deconvolution. The manuscript integrates clever UV–VIS study, centrifugation, absorbance changes, and particle size analyses to correlate ND concentration with different ND/DOX fractions, and potential in vitro biological relevance. The application of Weber–Moris intraparticle diffusion model allows distinction between different fractions—rapidly organized labile fractions and slower more firmly-bound ones—through a multi-step adsorption. Spectral deconvolution of UV–VIS spectra was used as a useful tool to announce the changes in different ND-engaged DOX species, which was not proposed before. The biological screening test on cells was performed giving the basic response; however, the results cover various cell lines thus providing broader insight, supporting the translational potential of ND as a drug carrier.

## 2. Materials and Methods

### 2.1. Preparation of the Solutions

The sample of DOX was prepared as follows: a certain volume of original solution (Doxorubicin Ebewe^®^ 50 mg/25 mL, EBEWE Pharma GmbH Nfg KG, Unterach am Attersee, Austria) was transferred into deionized water (WaterPro^®^ PS Systems, (Labconco WaterPro RO/PS Station, Kansas City, MO, USA)) to obtain a stock solution which was further used for the preparation of final solutions of DOX and nanoformulations (specified later in the text).

The aqueous solutions of nanodiamonds were prepared as follows: a certain volume of original solution (Sigma Aldrich-900185: Monodispersed nanodiamond particles 5 nm, 10 mg/mL in dimethyl sulfoxide-DMSO, Sigma-Aldrich, Merck KGaA, Darmstadt, Germany) was transferred into deionized water (WaterPro^®^ PS Systems) to obtain a stock solution which was further used for the preparation of final solutions of nanodiamonds and nanoformulations. DMSO content from the ND stock did not exceed 5% in the final samples applied to biological systems.

To minimize ND aggregation and DOX self-association, support component interaction, and to obtain stable aqueous dispersions, solutions were vortexed (LLG uniTEXER 1 Pro, LLG Labware, Meckenheim, Germany; ~5 s) and sonicated (Vabsonic VAB SB 4LDD, Vabsonic, Niš, Serbia; 240 W, 40 kHz, 10 min) immediately after the preparation and were kept in the dark at room temperature (21 °C) to prevent doxorubicin from light-induced degradation.

The series of concentration ratios ρ(ND)/ρ(DOX) [μg/mL]/[μg/mL] were prepared and investigated. ND/DOX samples had pH values ~6, without additional pH adjustment or buffer additives. Differences between preparation methods (vortex only vs. vortex + sonication) were preliminary evaluated and after a detailed spectral analysis, it was concluded that the sonication step could be omitted, which was important for the kinetic experiments where the technical reasons did not allow that procedural step. Nevertheless, subsequent experiments incorporated sonication into the preparation process whenever possible to ensure sample uniformity and reliability. Based on preliminary results, the most relevant ratios were selected for further experiments. In this study, only physical mixtures of DOX and nanodiamonds without any chemical functionalization or coupling agents to induce covalent bond formation were investigated. Therefore, the formation of covalently bonded ND-DOX species is not expected under these experimental conditions.

### 2.2. UV–VIS Spectroscopy Parameters and Protocol

UV–VIS spectra were measured using a Thermo Scientific Evolution Pro UV–VIS spectrophotometer (Thermo Fisher Scientific, Madison, WI, USA) with 1-cm quartz cuvettes. For kinetic experiments, the cuvettes were sealed with stoppers. Full spectral scans were performed within 350–650 nm, or absorbance was recorded at selected wavelengths. Deionized water was used as the blank. UV–VIS absorption spectra of doxorubicin (2.5 μg/mL) were recorded alone and in the presence of increasing concentrations (10–150 μg/mL) of nanodiamonds. After subtraction of the comparative ND spectra (10–150 μg/mL), the resulting difference spectra reflect the DOX-related spectral changes. In case of centrifugation: samples were measured before and after centrifugation, which was performed at 19,083× *g* and 4 °C for 1 h, with supernatant carefully removed for re-measurement. All procedures and sample storage performed in the dark (to the extent the technique allows it) to prevent light-induced changes.

The final mass concentration of ρ(DOX) = 2.5 µg/mL was chosen to allow the relevant biological applications in cell studies and UV monitoring, as it produces a clear and well-defined peak and is well below the proposed limit at which self-association of drug occurs [10,11,12]. In an initial set of experiments, ρ(DOX) = 0.5 µg/mL was also tested but proved unsuitable for reliable UV tracking under these experimental conditions. In parallel, the nanocarrier concentration was chosen to balance biological applicability with UV–VIS monitoring, keeping the levels low enough to minimize light scattering that could otherwise significantly compromise the interpretation of the analytical results. ND/DOX ratios were selected to cover a biologically relevant range for cell models acceptable for spectroscopic investigation, spanning minimal and maximal levels of each component to evaluate carrier and drug effects. This included combinations of low/high ND with low/high DOX, enabling assessment of minimal and maximal contributions of both components.

### 2.3. DLS/Zeta-Potential Measurements Parameters

DLS measurements and zeta potential (ζ) of nanoparticles in the investigated samples were analyzed by Zetasizer Nano ZS instrument (Malvern Instruments Ltd., Malvern, UK; 633 nm, 173-backscatter detection) in triplicates (size) and duplicates (zeta) in a disposable folded capillary cell DTS1060 at 25 °C. All samples were analyzed immediately after preparation and again after 24 h of storage in the dark.

### 2.4. Cell Lines Treatment

For the estimation of cell growth activity, three human malignant transformed cell lines-U251 (human glioma/astrocytoma cell line, EACC 09063001), HS294T (human melanoma cell line, ATCC HTB-140), and MCF-7 (breast adenocarcinoma, ECACC 86012803) and one non-malignant human cell line MRC-5 (fetal lung fibroblast, ECACC 84101801) were used. The cell lines were kindly donated by the collaborating Cell Culture Laboratory, Department of Histology and Embryology, Faculty of Medicine, University of Novi Sad, Serbia. To ensure continuity in working with the cell lines and to preserve their original characteristics, they were cryopreserved at –80 °C after every tenth passage and handled in accordance with ATCC and ECACC Animal Cell Culture Guidelines. The cell lines were grown and maintained in Dulbecco’s Modified Eagle’s Medium (DMEM, Sigma-Aldrich, St. Louis, MO, USA) supplemented with Fetal Calf Serum (FCS, 10%), penicillin (100 Units/mL) and streptomycin (100 μg/mL), being referred to as complete medium. The cells were cultured in 25 mL flasks at 37 °C in an atmosphere of 5% CO_2_ and high humidity, and sub-cultured twice a week. A single cell suspension was obtained using 0.1% trypsin with 0.04% Ethylenediaminetetraacetic acid (EDTA). Cells were plated into 96-well microtiter plates (Sarstedt, Newton, NC, USA) and pre-incubated in complete medium supplemented with 5% FCS at 37 °C for 24 h. Cells were cultured until they reached the logarithmic growth phase. Cell lines were treated for 48 h with five concentrations or combinations: (I) ND (10, 25, 50, 100, and 200 µg/mL), (II) DOX (1, 2.5, 5, 7.5, and 10 µg/mL), and (III) ND/DOX (µg/mL/µg/mL) combinations (50/1, 50/2.5, 50/5, 100/5, and 50/7.5). Cell viability was evaluated by the MTT (3-(4,5-dimethylthiazol-2-yl)-2,5-diphenyltetrazolium bromide) assay, and cytotoxicity (expressed as cell viability) is reported as the percentage relative to the untreated control (mean ± SD). All samples were analyzed in octuplicate (*n* = 8).

## 3. Results and Discussion

### 3.1. Possibilities of Determining Nanodiamond/Doxorubicin Interactions in the UV–VIS Region

Spectroscopic methods, especially in the UV–VIS range, are one of the most important tools in the study of nanodiamonds with various drugs [5,13]. Spectra could reveal the information on mutual interaction, and together with DLS analyses give an impactful insight into the interactions among nanodiamonds, as well as between nanodiamonds and drugs. By studying the UV–VIS spectra of ND/DOX combinations, we come to the marrow of the dilemma of these interactions—and we could set a question as to whether they induce mutual self-aggregation, or promote cooperative behavior within a newly-organized nanocarrier-drug system. In order to obtain the most accurate results and discussion it is essential to consider the following methodological limitations.

#### 3.1.1. Optical Properties of Nanodiamonds and the Selection of the Most Appropriate Wavelengths

One of the major limitations of UV–VIS spectroscopy in nanodiamond investigation is the optical properties of dispersed nanodiamonds—a delicate balance between two competing processes-light scattering and light absorption [14,15,16].

**Figure 1:**
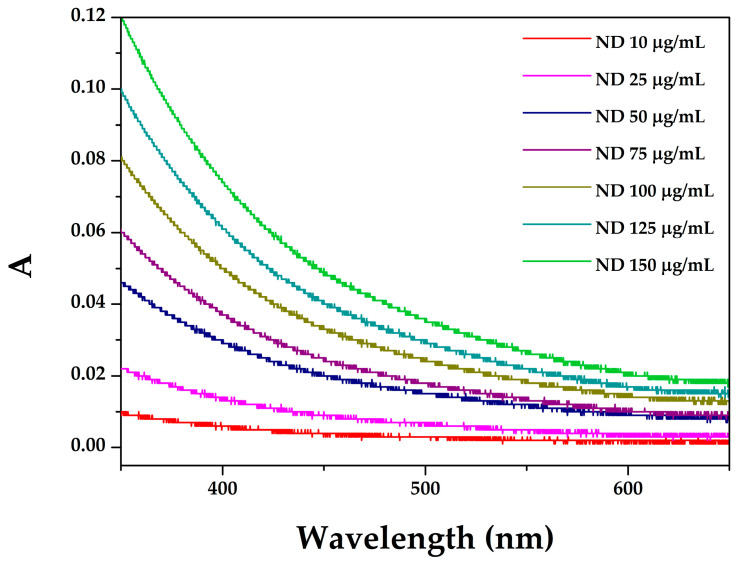
The spectra of a serial of nanodiamond concentration (10, 25, 50, 75, 100, 125, and 150 μg/mL, from bottom to top, respectively).

When nanoparticles size overgrows a certain size, scattering interferes with absorption spectra severely since it is strongly dependent on the size of particles. Therefore, staying in the frame of smaller particle size investigation, or simple purification/filtering of the samples, together with measuring at multiple higher wavelengths, will allow a quantitative determination without compromising the light absorbance. In this research, nanodiamonds originally used for the study are monodispersed nanodiamond particles of 5 nm, which when aggregated form the aggregates within 10–80 nm, mostly around 20 nm. The obtained spectra of a serial of nanodiamonds concentration (10–150 μg/mL) are presented in Figure 1. This figure shows a concentration-dependence without significant shifts or new emerging peaks, indicating stable optical properties and no observable aggregation within the tested concentration range.

The concentration of 50 µg/mL was selected as an optimal working point for the further kinetic examination because it provides a sufficient absorbance signal for reliable spectroscopic measurements maintaining linearity according to Lambert–Beer’s law.

**Figure 2:**
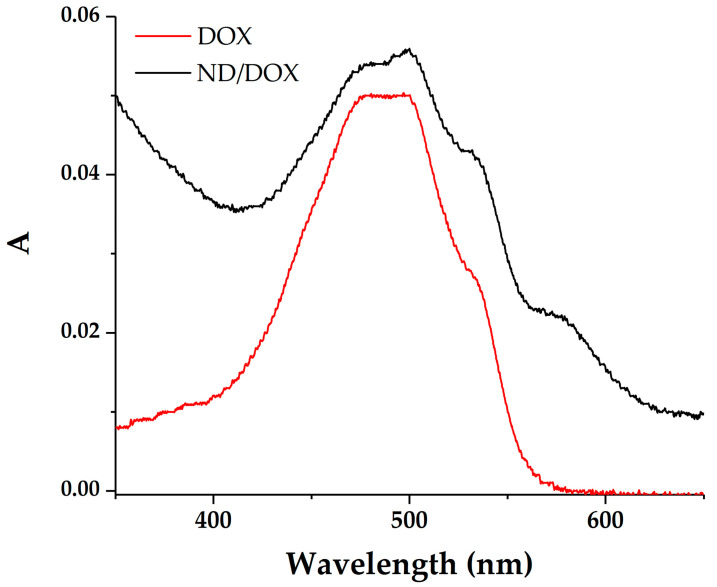
A preliminary spectroscopic scan of DOX (2.5 μg/mL) and ND/DOX combination (50 μg/mL/2.5 μg/mL) bottom to top, respectively, pointing at two prominent absorption peaks at the wavelengths of 500 and 580 nm.

The preliminary scans (Figure 2) of DOX and the ND/DOX combination revealed two distinct peaks at 500 and 580 nm, which were selected to investigate interactions and to monitor the complex stability under both static (equilibrium) and dynamic (kinetic) conditions.

**Figure 3:**
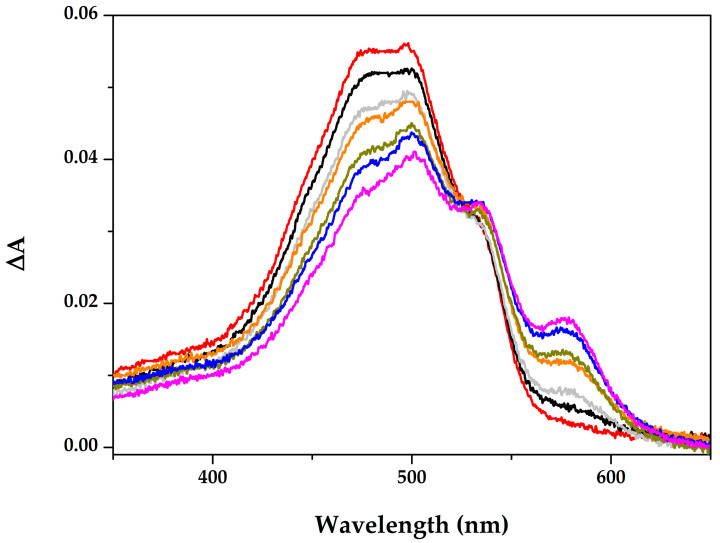
The resulting difference spectra of subtracted corresponding ND spectra from ND/DOX spectra. DOX concentration is constant in all ND/DOX samples, ND concentration is increasing from top to bottom (10, 25, 50, 75, 100, 125, and 150 μg/mL).

The wavelength of 580 nm seems optimal for studying the ND/DOX interaction. At this wavelength, free monomeric DOX shows minimal absorbance, while nanodiamonds show negligible absorbance and minimal contribution from scattering. Therefore, the changes in absorbance at 580 nm could be attributed predominantly to ND/DOX interaction. On the other hand, analysis at 500 nm requires additional consideration, as both light scattering by nanodiamond aggregates and deviations from the Lambert–Beer law additivity may influence the measured absorbance. Difference spectra obtained by subtracting the corresponding ND spectra from ND/DOX spectra over a series of concentrations are shown in Figure 3.

The spectra reveal two prominent trends. First, as the ND concentration increases, the intensity of the dominant DOX maximum absorption band at 480–500 nm gradually decreases (hypochromic effect) corresponding to the π–π transitions of the anthracycline chromophore, indicating the decrease in free DOX concentration, most likely due to DOX adsorption onto the ND surfaces [13]. At the same time, reshaping of the signal occurs—a feature at ~580 nm becomes more pronounced (hyperchromic effect), which is not observed for DOX alone and may reflect the formation of ND-associated DOX species. The ~580 nm peak represents a red shift, primarily reflecting changes in the electronic structure of doxorubicin. Doxorubicin contains a planar, conjugated aromatic system with quinone moieties acting as hydrogen-bond donors and acceptors. Such a system is sensitive to alterations in its electronic structure, both at the molecular level (through self-association, complexation, or donor-acceptor interactions) and due to changes in the surrounding environment (refractive index and dielectric permittivity). Importantly, since this peak is absent in the spectra of pure doxorubicin, appearing only in the presence of nanodiamonds, we approximate this feature as the “ND–DOX bound form” without attempting to deconvolute additional potential effects that cannot be clearly separated thermodynamically, experimentally, or theoretically.

The isosbestic point at 530 nm most likely reflects the coexistence of free and bound DOX, without degradation, as similar environment-dependent spectral changes have already been described for this drug [17,18]. A shoulder at 540 nm remains slightly changed with increasing ND concentration and likely represents free or weakly aggregated DOX molecules not directly involved in interactions with NDs. The authors have focused on the 480–500 nm and 580 nm peaks as the most dynamic. Overall, an increasing concentration of ND suppresses DOX absorbance and changes the spectrum shape, which may be the result of the following mechanistic: π–π interactions with ND, formation of ND/DOX charge–transfer complexes, changes in polarity or rigidity near the chromophore that come from the interactions with carboxyl and hydroxyl groups or sp^2^-species from ND, or even DOX–DOX self-aggregation [5,19,20].

**Figure 4:**
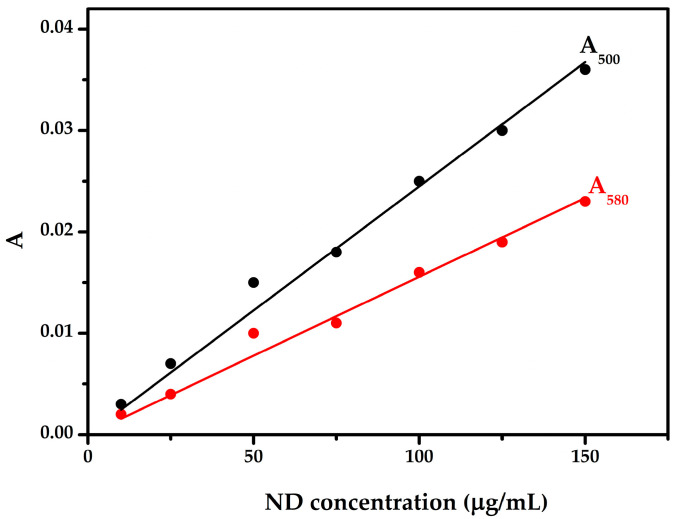
Absorbance of nanodiamonds as a function of concentration at 500 nm (A_500_, black) and 580 nm (A_580_, red). The linear fits were used to determine the extinction coefficients according to the Lambert–Beer law.

Linear fits to the absorbance-concentration dependence of nanodiamonds were used to determine the extinction coefficients (Figure 4) presenting good linearity within the investigated concentration range. Within the validity of the Lambert–Beer law, the extinction coefficients of nanodiamonds at 500 and 580 nm were determined to be 0.0002 and 0.00015 cm^2^·µg^−1^, respectively.

Taken together, the additivity principle of the Lambert–Beer law, supported by measurements at multiple higher wavelengths, indicates that UV–VIS examination is a valid approach for investigating non-covalent interactions between nanodiamonds and drugs.

#### 3.1.2. Additivity of the Lambert–Beer Law and the Influence of Centrifugation

To address the question of whether, and within which concentration range, the ND/DOX interaction can be reliably studied without centrifugation, and whether linearity of absorbance with concentration can be preserved under such conditions, a series of ND and ND/DOX mixtures were examined at 500 nm (before and after the centrifugation).

**Figure 5:**
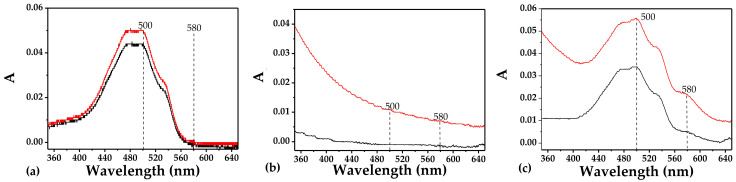
Effect of centrifugation on the absorbance spectra of (**a**) DOX (2.5 μg/mL), (**b**) ND (50 μg/mL) and (**c**) ND/DOX combination (50 μg/mL/2.5 μg/mL). The upper curves correspond to spectra acquired prior to centrifugation.

The following final concentration ratios ρ(ND)/ρ(DOX) [μg/mL]/[μg]/mL] were investigated: 10/2.5, 50/2.5, 100/2.5, and 150/2.5. For each mixture, full spectra were analyzed with and without centrifugation and some of the results are presented in Figure 5. This figure illustrates the effect of centrifugation on the absorbance for the following samples: pure DOX (2.5 μg/mL), pure ND (50 μg/mL), and ND/DOX (50 μg/mL/2.5 μg/mL).

**Figure 6:**
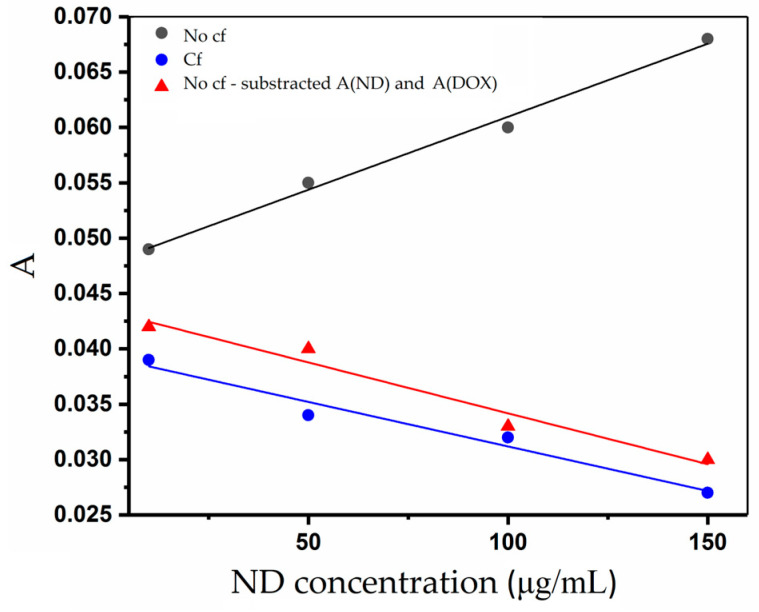
Theoretical verification of Lambert–Beer law additivity at 500 nm before (no cf) and after (cf) centrifugation for different ND/DOX ratios.

Figure 6 demonstrates that, after centrifugation, linearity consistent with the additivity of the Lambert–Beer law is preserved across all investigated ND/DOX ratios. The nanodiamond contribution to absorbance was theoretically subtracted using the previously determined extinction coefficient of nanodiamond at the corresponding concentration.

These results indicate that centrifugation is not essential for reliable spectroscopic evaluation within the examined concentration range. Nevertheless, to gain deeper insight into the ND/DOX interactions, and to map the possible steps in complex formation, centrifugation and kinetics experiments were conducted.

#### 3.1.3. Spectral Deconvolution and Stability of ND-Induced DOX Complexes: Redistribution and Binding Heterogeneity

To properly analyze the relative distribution of different DOX species, their involvement in binding, as well as ND/DOX interactions, it is essential to consider the species present in the samples. Besides free DOX and ND, several differently stable fractions of ND/DOX coexist. DOX alone undergoes self-association through multiple equilibria, which strongly depend on concentration, surrounding species, and intermolecular interactions [21]. Spectroscopically, doxorubicin is characterized by a complex absorption band that could be represented as superposition of several simpler bands, corresponding to DOX species with different electronic distributions. A closer inspection of the DOX spectrum reveals lower-wavelength forms, predominantly associated with free DOX, and higher-wavelength forms, attributed to bound forms of DOX [11,22,23,24]. This distribution is rather dynamic and the result of a balance between DOX, DOX–DOX, and DOX–ND interactions. To investigate DOX involvement in ND/DOX interactions, a series of samples with a constant initial DOX concentration of 2.5 μg/mL and increasing ND concentrations (10–150 μg/mL) were prepared. For each mixture, full UV–VIS spectra were recorded prior and after centrifugation. Nanodiamond background absorbances were subtracted from the corresponding ND/DOX absorbances.

##### Spectral Deconvolution and Redistribution of DOX Species

Gaussian deconvolution of the spectra revealed four absorbing species, whose relative contributions change with increasing ND concentration. *Savitzky–Golay* smooth digital filter is used for signal noise reduction. For the spectral deconvolution, manual fitting were used assuming four Gaussian functions in the range within 350–650 nm. For all spectral treatments *Fytik* software was used (https://fityk.nieto.pl/, accessed on 15 January 2026). The reported frequencies and half-widths were reproducible within 0.2 and 1 nm.

**Figure 7:**
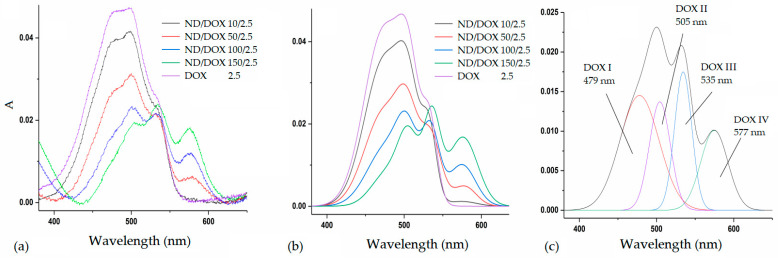
(**a**) Pure DOX and ND/DOX raw absorption spectra, (**b**) theoretically modelled DOX and ND/DOX absorption spectra, (**c**) pre-assigned absorbing species from the deconvoluted spectrum of the ND/DOX.

Figure 7 shows the evolution of DOX absorption bands upon gradual addition of ND. The band centered at approximately 479 nm (DOX I) corresponds to free, unbound DOX molecules. The band at ~505 nm (DOX II) is assigned to weakly bound self-associated DOX species, most likely dimers and trimers. The band at ~535 nm (DOX III) corresponds to more strongly bound DOX–DOX dimers, presumed to form larger self-organized clusters. Finally, the band observed at ~577 nm (DOX IV) is most likely attributed to DOX bound to nanodiamond, as it is absent in the spectrum of pure DOX and emerges only upon ND addition (i.e., increases with the increase in ND concentration).

**Figure 8:**
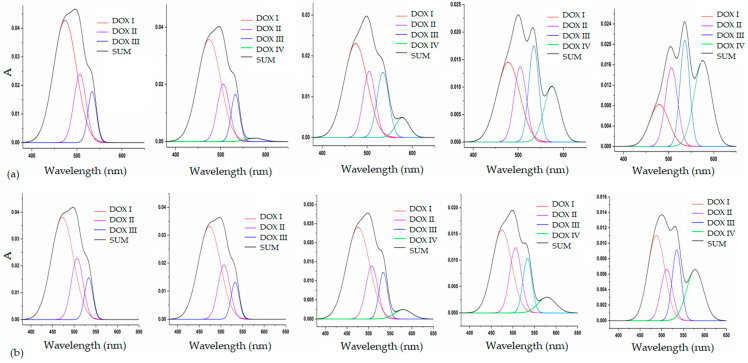
Shift in the equilibrium between free and self-associated DOX species with increasing nanodiamond concentration in ND/DOX mixtures (**a**) before centrifugation, (**b**) after centrifugation.

Analysis of the deconvoluted spectra demonstrates a pronounced redistribution of DOX species with increasing ND concentration. The contribution of free DOX (DOX I) decreases most strongly, while weak self-associates (DOX II) decline to a lesser extent. Hypochromic effect might indicate increased π–π stacking between antraquinone system form DOX and sp^2^-species on the NDs surface. On the other hand, the contribution of bound DOX species increases. The DOX III band (multimer) initially shows a slight decrease followed by a slight increase, while the DOX IV band shows a continuous and intensive increase. This behavior provides strong evidence that the band at ~577 nm corresponds to the formation of ND-induced DOX complexes. The intensive shift from free to bound DOX species before (a) and after (b) centrifugation is further emphasized and presented in Figure 8.

Detailed deconvolution parameters, the absorption maxima positions and band full widths at half maximum (FWHM), are listed in Table 1.

**Figure 9:**
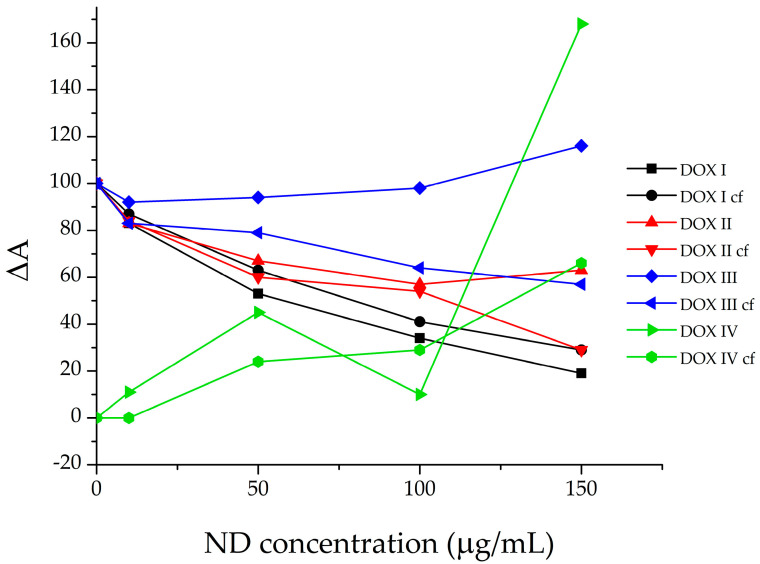
Comparative overview of the ratios of different doxorubicin forms in the presence of increasing nanodiamond concentrations, before and after (cf) centrifugation.

When the relative intensities of the individual bands are presented graphically (Figure 9), the shift of the equilibrium from free toward bound forms of doxorubicin with increasing nanodiamond concentration becomes clearly evident.

The data suggest that the amount of free DOX decreases proportionally with the increase in ND concentration. A similar pattern is observed after centrifugation. Comparable trends are found for DOX II and DOX III species, indicating that increasing ND concentration induces a reorganization of DOX dimmers/multimers to re-establish the disturbed equilibrium by engaging free DOX into ND-induced DOX complexes. This may indicate the reorganization of DOX-DOX stacking facilitated by nanodiamond surfaces, or even indicate anchoring of DOX molecules within nanodiamond aggregates. This insertion may promote more ordered and constrained orientation of nanodiamond-bound DOX molecules, thus facilitating more efficient further interactions. In contrast, the trend observed for ND/DOX (DOX IV) follows a different pattern, and most likely corresponds to complex formation of charge–transfer character. The initial interaction between ND and DOX IV appears to occur at ND concentrations up to 50 μg/mL, followed by a slight decrease, and then a pronounced increase at ND concentrations above 100 μg/mL. Spectral deconvolution after centrifugation reveals a proportional increase that could be attributed to the fraction of ND/DOX remaining stable in the supernatant after centrifugation.

##### Centrifugation as a Probe of ND/DOX Binding Stability

Centrifugation was employed as a mechanical test to differentiate ND/DOX based on their binding stability and spectral response.

**Figure 10:**
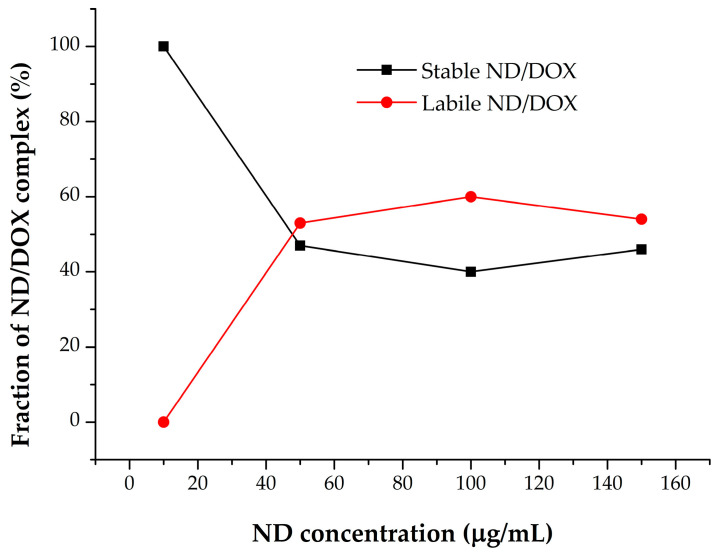
Relative contributions of labile and stable ND/DOX as a function of ND concentration.

Prior to centrifugation, DOX is present as: DOX (monomeric or associated species), characterized by the band at ~470–480/500/535 nm; weakly bound DOX to ND; and stably bound ND/DOX. After centrifugation, weakly bound DOX either detaches from the ND surface or co-sediments within larger unstable ND aggregate. On the other hand, stabile ND/DOX complexes, depending on their size and colloidal stability, can either remain in supernatant (if small and colloidally stable) or sediment. The probable evidence for the formation of stable ND/DOX is reflected in the residual post-centrifugation absorbance signal at 580 nm. Absorbance at 580 nm (A_580_) was therefore used to differentiate ND/DOX complexes according to their binding stability, while absorbance at 500 nm (A_500_) was employed to quantify DOX removal from solution. The results are summarized in Table 2 and visualized in Figure 10.

At low ND concentrations, the majority of DOX seems to be engaged within the stable ND/DOX complexes. Following the increase in ND concentration, ΔA_580_ increases as well, thus indicating the increased fraction of labile ND/DOX complexes removed by centrifugation. This trend suggests that stable binding sites seem to approach saturation at higher ND concentrations. When changes in A_500_ follow changes in A_580_, this indicates proportionality between free DOX removal and DOX involved in ND-induced DOX complexes formation. The combined use of A_580_ and A_500_ proposes a heterogeneous binding mode, consisting of a stable core ND/DOX and an increasingly dominant labile component at higher ND concentrations (Figure 10). These findings correlate well with the spectral deconvolution analyses of the investigated samples.

#### 3.1.4. Dynamic Character and Kinetic Modelling of the ND/DOX Interaction

The standard procedure for preparation of ND/DOX complexes involves mixing the components and allowing the system for 24–48 h to establish equilibrium prior to analysis or application [6]. However, the interactions between nanodiamonds and drugs are complex, involving covalent and non-covalent reactions, agglomeration, as well as adsorption and diffusion phenomena. Kinetic experiments can provide valuable insights into these processes. Although these interactions often occur rapidly, careful experimental determination of the kinetics, followed by modeling, can reveal important information about the factors that limit the reactions. Accordingly, in this study we aimed to determine the reaction kinetics of nanodiamonds with doxorubicin and to assess whether the process is primarily diffusion- or reaction-controlled.

**Figure 11:**
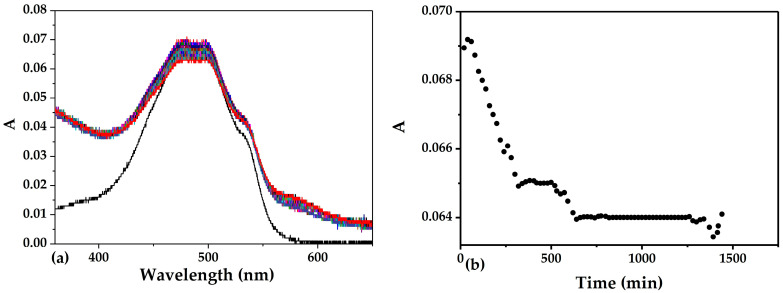
(**a**) Time-dependent UV–VIS absorbance spectra of DOX the ND/DOX recorded over 24 h, (**b**) Reaction kinetics of the ND/DOX system over 24 h, showing changes in absorbance at 500 nm.

In this work, the kinetics of the ND/DOX interaction was investigated over two distinct time frames. Long-term kinetics were monitored by recording the absorbance of the complex at 500 nm over 24 h every 20 min (Figure 11).

Analysis of the 24 h kinetic profile reveals that, after an initial fast phase, the system shows minimal changes in absorbance. The dominant interaction processes seem to occur within the first seconds to minutes after mixing.

**Figure 12:**
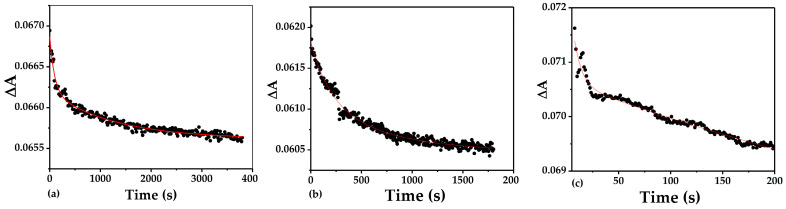
Short-time kinetics of the ND/DOX interaction monitored at (**a**) 15 s (1 h), (**b**) 5 s (30 min), and (**c**) 1 s (3 min) intervals at 500 nm.

To further analyze this rapid reaction, the early-stage kinetics were examined by monitoring changes in doxorubicin absorbance under the following conditions: 1-h kinetics with a 15 s interval, 30-min kinetics with a 5 s interval, and 3-min kinetics with a 1 s interval (Figure 12).

The obtained experimental data were fitted with different models. The best agreement was obtained with a biexponential decay function of the form, where A_1,_ A_2,_ *t*_1,_ *t*_2_ represent the fit coefficients of exponential model.

(1) $$y=A_{1}e^{\frac{x}{t_{1}}}+A_{2}e^{\frac{x}{t_{2}}}+y_{0}$$

A good fit with the biexponential decay function indicates multi-stage adsorption, combining rapid surface adsorption and slower “pore” diffusion. The fast kinetic is dominant within the first seconds of the interaction when the drug rapidly binds to accessible surface sites on nanodiamonds. Here, this phase can be likely associated to the electrostatic or charge–transfer-driven adsorption of doxorubicin onto exposed nanodiamond surface functional groups. On the other hand, the slower phase could include infiltration of doxorubicin within the nanodiamond aggregates, or nanodiamond-induced rearrangement of doxorubicin or vice versa.

**Figure 13:**
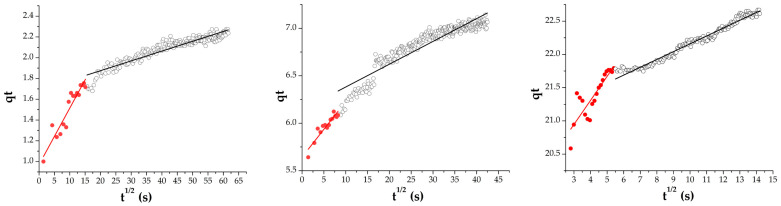
Adsorption data fitted according to the Weber–Morris intraparticle diffusion model.

The previous kinetic plots reveal the overall adsorption rate and the best fit of certain model. However, to dive into the mechanism which explains in more detail how doxorubicin moves within nanodiamonds (intraparticle diffusion), and if it is the rate-limiting step, one should apply the Weber–Morris plot (Figure 13)

(2) $$q_{t}=k_{id}\sqrt{t}+c$$

where *c* is the concentration on the adsorbent (μg/mg) at time *t*, *k_id_* is the intraparticle diffusion rate constant (μg/mg min^0.5^), and *C* is a constant related to the boundary layer thickness.

According to the theoretical framework of the employed kinetic model, if the plot passes through the origin, the kinetics are entirely controlled by intra-particle diffusion. If this is not the case, a multi-step mechanism could be proposed. The initial steep slope corresponds to the rapid surface adsorption, while the following region corresponds to gradual drug infiltration within nanodiamonds. Several authors have noted that the intercept values, as well as the slopes of multiple linear regions within the model, suggest that the kinetics are influenced not only by internal but also by external diffusion, during which a boundary diffusion layer, film, forms around the adsorbent particles. The thickness of this layer creates a concentration gradient responsible for mass transport. In our study, the kinetic experiment was conducted by adding a nanodiamond solution to a doxorubicin solution at the initial moment. At this stage, the nanodiamond-to-doxorubicin ratio is very low, and the rapidly adsorbed labile fraction dominates, corresponding to the intercept at the graph. During this initial period, less organized self-assembled aggregates of both nanodiamonds and doxorubicin, each with their respective particle size distributions, interact and form surface films that control the early stage of adsorption. In this phase, intra-particle diffusion has minimal impact, as larger, more organized, and stable aggregates have not yet fully formed. With the progress of reaction, reorganization occurs; DOX molecules reorganize within growing ND aggregates leading to the formation of more stable and larger aggregates. The stable fractions then dominate the adsorption process through intra-particle diffusion (the second phase described by the Weber–Morris model as a linear region). This behavior is consistent with previous studies on adsorption kinetics, including the removal of color using various adsorbents [25], tetracycline adsorption on H_3_PO_4_-activated carbon [26], and sulfur removal in oxidative-adsorptive desulfurization [27], where multi-step diffusion mechanisms and boundary layer effects were similarly observed.

The results of spectra and centrifugation-induced changes analyses further support this mechanism. The decrease in free DOX concentration with the increase in ND concentration indicates that ND surfaces promote reorganization among DOX dimers and multimers into ND-bound complexes in such a way that the labile fraction is the first one to establish, with the ratio of labile and stable ND/DOX complexes depending on ND concentration. At low ND concentrations, DOX predominantly forms stable ND/DOX complexes, while the dominance of the labile fraction is proposed at higher ND concentrations. These observations contribute to the heterogeneous binding model, consisting of a stable ND/DOX core and variable labile component. Altogether, these findings suggest that DOX adsorption onto ND is controlled by a combination of surface interactions (labile fraction) and intra-particle diffusion (stable fraction). The combination of kinetic experiments, spectroscopic data, and centrifugation results together highlight the potential of nanodiamonds as stable carriers for doxorubicin, combining rapid initial uptake with sustained adsorption over time.

Although the usual preparation protocol allows 24–48 h for equilibration, the kinetic data clearly demonstrate that the equilibrium is effectively reached much earlier, within the first minutes after mixing, when the absorbance significantly decreases, after which it reaches the plateau and remains fairly constant over the remaining 24 h. Therefore, an extended incubation time ensures the stable fraction completes intra-particle diffusion, although the labile fraction reaches equilibrium within minutes.

### 3.2. DLS Measurements of ND and ND/DOX Samples

Dynamic light-scattering measurements were conducted to evaluate the particle size distribution and zeta potential of ND and ND/DOX samples. The samples were measured immediately and after a 24 h incubation to monitor potential aging effects and assess short-term sample stability.

#### 3.2.1. Effect of ND Concentration and Aging on Particle Size Distribution and Zeta Potential

**Figure 14:**
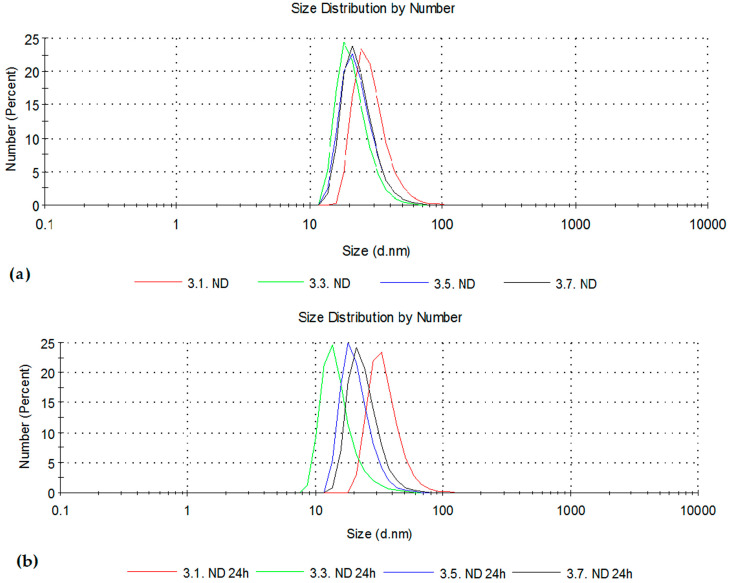
Size distributions (by number) of nanodiamond suspensions at 10, 50, 100, and 150 µg/mL measured by dynamic light scattering (**a**) immediately after preparation, (**b**) after 24 h of incubation.

ND dispersions at concentrations 10 (3.1), 50 (3.3), 100 (3.5), and 150 (3.7) μg/mL were analyzed immediately after preparation and after 24 h. The corresponding particle size distributions by number are shown in Figure 14a,b. One should take into consideration that size distribution by number is highly sensitive to small particles.

The particle size distributions by number revealed a dominant particle population within the 10–80 nm range, with the majority around 20–30 nm. Minor variations between repeated measurements were more pronounced at lower concentrations, which can be attributed to reduced scattering intensity and increased sensitivity of DLS measurements in dilute dispersions [28]. Neither significant aggregation nor broadening of the particle size distribution was observed with the increase in ND concentrations, which suggests that the colloidal stability of ND dispersions was preserved within the investigated concentration range.

In comparison to the literature data reporting a zeta potential of approximately +53 mV [6], the values obtained in this research show slightly lower values; however, comparable and within the same positive trend. With increasing ND concentration, a slight increase toward more positive zeta potential values was observed. The average zeta potential values for ND concentrations of 10, 50, 100, and 150 μg/mL were approximately +10, +20, +25, and +28 mV, respectively. The observed trend may be related to improved reliability in the samples of higher ND concentration. However, the values indicate electrostatic contribution to overall colloidal stability.

After 24 h, particle size distributions remained similar and predominantly uniform for all concentrations, without signs of aggregation over time. Similarly, zeta potential values remained relatively stable, with average values of approximately +20, +23, +24, and +26 mV for ND concentrations of 10, 50, 100, and 150 μg/mL, respectively.

#### 3.2.2. ND/DOX Systems: Immediate Characterization and the Effect of Aging

**Figure 15:**
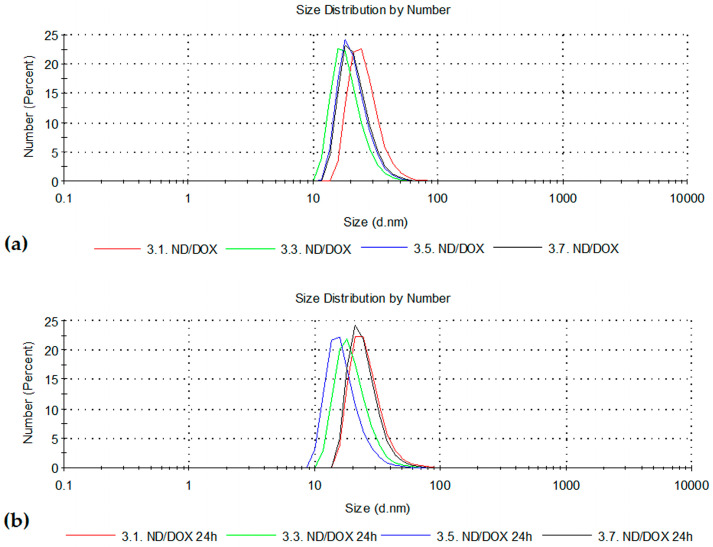
Size distributions (by number) of ND and ND/DOX at ND concentrations of 10, 50, 100, and 150 µg/mL with constant DOX concentration of 2.5 µg/mL, measured by dynamic light scattering (**a**) immediately after preparation, (**b**) after 24 h of incubation.

The effect of doxorubicin loading on ND was evaluated by comparing ND dispersions (10, 50, 100, and 150 μg/mL) with corresponding ND/DOX mixtures, while the DOX concentration was kept constant at 2.5 μg/mL. The results are presented as size distributions by number in Figure 15a (immediately after preparation) and Figure 15b (after 24 h of incubation).

Immediately after sample preparation, the particle size distribution in ND/DOX remained comparable to those in corresponding ND dispersions, with a very slight shift toward smaller values in ND/DOX samples (on the order of a few nm). The distributions were uniform, without significant changes in particle size or signs of aggregation. Unlike these results, literature data suggest the increase in hydrodynamic size after nanoparticle modification with DOX from 35 nm to 51 nm [6]. Our current research also points out that the particle distribution was more uniform at higher concentrations. Namely, although distributions and general trends were similar overall, differences between repeated measurements were almost negligible. In corresponding ND-only samples, a certain degree of non-uniformity in particle size measurements was observed, which was not present in ND/DOX samples, suggesting that DOX may contribute to improved colloidal homogeneity in this experimental setup.

The aggregation behavior of carbon-based nanomaterials, such as nanodiamonds, is highly sensitive to sample concentration, preparation, and the presence of adsorbed molecules. Uniform sample preparation, including sonication, is essential in order to minimize the variability and ensure reproducible DLS measurements. Analysis of the particle size data given in the table below indicates that ND and ND/DOX dispersions exhibit a concentration-dependent trend. At lower concentrations, the variability between repeated measurements is greater as SD indicates which can be attributed to reduced scattering intensity and increased sensitivity of DLS in dilute dispersions. In contrast, higher concentrations yield more uniform and stable dispersions most probably due to hindered Brownian motion and limited aggregate rearrangements. The differences in the uniformity were reflected in the SD of the performed measurements, as well as in the values of the values polydispersity index (PDI). The preview of average sizes with SD values is given in the Table 3.

Presenting the results via a number-based particle size distribution curve offers a true reflection of the particle size distribution measured in the analyzed solution, with clearly differentiated fractions of different sizes. In this experiment, it indicates that the majority of particles are within 10–80 nm, with peaks around 20–30 nm, whereas average values range between 45–105 nm due to the presence of minor fractions of larger particles that shift the result towards the larger values. This highlights that the average size alone may be misleading, while the number-based particle size distribution curves more accurately represent the true distribution and distinct particle fractions.

The heterogeneity in samples generally has significant biological relevance; samples with broader distributions can give variable uptake: smaller particles may enter the cells more facile, while larger fractions could contribute to controlled or sustained release of the drug, or not enter the cell at all. It is well known that differently sized particles follow different entry pathways to the cell, thus the differences in size within a single nanoformulation could potentially provide multiple attack to the malignant cells.

Zeta potential values of approximately 20, 24, 25, and 29 mV were observed for the following ND/DOX samples 10/2.5, 50/2.5, 100/2.5, and 150/2.5 ND/DOX [μg/mL/μg/mL], respectively. These values are comparable to those obtained for pure ND dispersions, suggesting that the DOX decoration does not significantly alter the overall surface charge of nanodiamonds and that DOX only partially screens the nanodiamond surface. The similar ND and ND/DOX zeta potentials also suggest that adsorption is mostly driven by interactions other that electrostatic. The literature data, on the other hand, reported a decrease in zeta potential observed for drug-functionalized ND in comparison to pure ND [6,29,30], with preserved colloidal stability.

After 24 h, the mean zeta potential values of ND/DOX samples were approximately 23, 28, 30, and 28 mV. With aging, no major changes in zeta potential values were observed within the tested ND/DOX samples. Slight shifts or deviations in zeta potential measurements, similar to those observed for particle size, were most noticeable in samples with the lowest ND concentrations, where the influence of DOX was more pronounced due to the relatively low ND concentration and where the measurement sensitivity is higher. Overall, after 24 h of aging, ND/DOX samples showed minimal changes in both particle size distribution and zeta potential, indicating that the systems remain colloidally stable over time.

The polydispersity index was further evaluated in order to assess the homogeneity of the investigated systems. For ND dispersions, immediately after preparation, the lowest concentration exhibited a PDI of about 0.2, followed by a slight increase and subsequent decrease at higher ND concentration. ND/DOX systems showed a similar trend. In ND/DOX samples, an increase up to 0.3–0.4 was observed in the first two samples, but with increasing ND concentration in ND/DOX, PDI shifted toward lower values of around 0.2. This behavior could suggest that in lower ND-containing systems the contribution of DOX to the particle surface and organization is more pronounced, while the increase in ND concentration contributes to more uniform system. PDI values between 0.3–0.4 refer to the moderate polydispersity. Upon aging, in ND dispersions PDI shifted to approximately 0.3–0.4, except for the ND sample with the highest concentration, where PDI was slightly below 0.2. In ND/DOX samples (10/2.5, 50/2.5, 100/2.5, and 150/2.5 [μg/mL/μg/mL]), PDI values were approximately 0.4, 0.3, 0.3, and 0.2, respectively. During aging, DOX appears to contribute to dispersion uniformity under the examined experimental setup.

For further biological application, the key parameter is the particle size range of both ND and ND/DOX, which in all tested samples remained below 80 nm, which is within the range often considered favorable for the optimal cellular uptake [31] and potentially suitable for the in vivo potential passive tumor accumulation via the enhanced permeability and retention effect. Considering the negatively charged cell membranes surface, the positive zeta potential of the tested samples may be advantageous for efficient cellular uptake due to attractive electrostatic forces between oppositely charged cell surfaces and nanoparticles [32,33]. The confirmed colloidal stability of the examined systems greatly contributes to the safety and reproducibility of experimental models for further research on nanodiamond-based drug delivery systems.

### 3.3. Biological Validation

To the best of our knowledge, this study represents one of the first comparative evaluations of ND/DOX systems across melanoma (HS294T), glioblastoma (U251), and breast cancer (MCF-7) cell models. Moreover, the inclusion of non-malignant cell lines enables the comparison of the effect of ND/DOX toward malignant cells versus non-malignant cells (MRC-5). The most prominent results are summarized in Table 3.

Based on the obtained experimental results, nanodiamonds alone (10–200 µg/mL) exhibited relatively low cytotoxicity (78–94% viability) without a clear concentration-dependent response in all tested cell lines. This outcome is aligned with the literature data [34,35,36,37,38]. Similar response and trend (approx. 80–88% viability, no clear concentration-response) was observed in normal fibroblasts (MRC-5) suggesting altogether good biocompatibility and potential safety of an ND-based drug delivery system [38,39,40].

As for doxorubicin, a clear concentration-dependent decrease in cell viability with increasing DOX concentrations (1–10 µg/mL) was observed in all examined cell lines. The strongest cytotoxic effect (at 10 µg/mL) was observed in HS294T and U251 (viability approx. 41%), while in MCF-7 and MRC-5 it was approx. 53% and 60%, respectively. At the lowest concentration (1 µg/mL), cell viability was around 78% (U251), 87% (MCF-7), 81% (HS294T), and 74% (MRC-5) compared to control. At 2.5 µg/mL, viability drops sharply to approximately 54% in the case of the U251 cell line, while in the other cell lines it changes to 66% (MCF-7 and HS294T), and 72% (MRC-5). Based on the obtained results, it could be suggested that, within the investigated range, the MRC-5 cell line appears less dose-sensitive. These changes pointed to the concentrations that would be the most interesting for further investigation and result discussion. A further increase in DOX concentration resulted in incremental concentration-dependent decrease in cell viability. The highest sensitivity of U251 towards DOX treatment may be the result of its high proliferative index [41] which is strongly influenced by DOX (DNA intercalation, topoisomerase II inhibition). In addition, DOX-induced ROS generation may additionally increase this cell line vulnerability.

ND/DOX combinations resulted in vaguely cell line-specific responses. The representative effects of ND, DOX, and ND/DOX on cell viability after 48 h are given in Table 4. In the HS294T cell line, ND/DOX combinations slightly enhanced cytotoxicity in comparison to the sole drug. Based on a cell viability comparison, the most effective response was observed for ND/DOX (50/7.5), reducing viability to approximately 37% compared to DOX alone (approx. 47%). The results of the Bliss model analyses presented in Table 5 suggest a synergistic effect for the ND/DOX combinations 50/2.5, 50/5, and 50/7.5 in the HS294T cell line. Similar analyses were performed for U251, MCF-7, and MRC-5 cells, with no synergism observed. A similar, but less pronounced effect was observed for ND/DOX (100/5) resulting in cell viability around 50%.

In U251 cell line combinations, ND/DOX in comparison to DOX alone showed a similar trend and negligible differences. Although the treatment with ND/DOX (50/7.5) resulted in the lowest viability (approx. 42%), in comparison to ND/DOX (50/2.5) the difference was minimal, suggesting limited benefit from the application of higher DOX concentrations. Therefore, ND/DOX (50/2.5) may represent an optimal combination balancing the potentially safer treatment and the adequate effect.

In the MCF-7 cell line, ND/DOX combinations generally did not show any significant enhancement in comparison to DOX alone. The highest cytotoxicity was observed for the combination 100/5 and 50/5 (viability approx. 59%, and 57%, respectively) without any indications of potential drug-carrier synergism.

The MRC-5 cell line response shows the moderate cell viability reduction upon ND/DOX treatments (approx. 56% in the case of ND/DOX 100/5) with no indications of the potential drug-carrier synergism. MRC-5 cells seem to be generally less sensitive to DOX and ND/DOX combinations in comparison to malignant cell lines, indicating potentially slight selectivity for cancer cells. The fact that ND does not induce enhanced toxicity in comparison to a drug alone may be a solid starting point for a potential improvement in the therapeutic index.

Representative concentrations (Table 4) were selected to enable assessment of the potential cell line-specific response and optimal treatment rather than maximal cytotoxic effects.

The rationale behind the chosen concentrations/combinations is the following: ND (50 µg/mL) presents the mid-range concentration that exhibits the minimal/average cytotoxicity across all cell lines. Therefore, any significant reduction in viability after ND/DOX treatment will not be attributed to ND. DOX (2.5 µg/mL) is the inflection point, not too mild or too toxic, at which cell viability in U251 drops sharply—efficient to monitor whether the nanocarrier improves drug performance at submaximal doses. ND/DOX (50/2.5) exhibits minimal additional toxicity in MRC-5, while results in different responses in malignant cell lines remain.

Different cell responses may be associated with differences in metabolism, oxidative stress, and protective systems among the cell lines [44], underlining the importance of the biological environment and experimental model. Doxorubicin-induced cytotoxicity is strongly associated with oxidative stress and increased reactive oxygen species formation in cells. Bearing this in mind, the differences in redox balance and stress-response capacity between non-malignant MRC-5 fibroblasts and tumor cell lines may partly explain the distinct sensitivity patterns observed in vitro, as MRC-5 cells frequently exhibit lower susceptibility under comparable treatment conditions [45,46]. In the case of MCF-7, drug efflux and slower proliferation could be the reason for the relatively modest response to the treatments conducted within this experimental setup, whereas ND-DOX formulations are reported to enhance cellular delivery/retention and can partly bypass efflux-based resistance mechanisms [47,48].

The use of ND as a drug carrier was expected to potentially allow the application of lower doses of DOX, thereby reducing systemic toxicity and making this therapy more effective and safer. The particle size and stability could significantly impact the efficiency of cellular uptake of ND/DOX complexes, thus potentially contributing to a more effective treatment. The fact that the majority of the particles investigated in this research were around 20 nm is particularly promising for future investigations [13,49,50] and suggests a possible high cellular uptake efficiency without being cleared too quickly by the reticuloendothelial system (RES). In addition, these particles tend to accumulate in tumors more effectively, increasing the potential for targeted drug delivery. Strong ND-induced DOX complexes (DOX IV) likely represent the “core” of ND/DOX particles internalized by cells, while labile ND/DOX could be those that more easily release DOX suggesting the potential multi-level controlled drug release. Depending on how ND influences the DOX aggregation or complexation, it could either enhance or reduce the drug efficacy. Aggregation might reduce the drug’s availability, potentially decreasing its cytotoxicity. On the other hand, if ND stabilizes DOX in some of the forms, in cellular uptake or delivery this could improve its therapeutic potential. Further studies are necessary to validate these findings and contribute to a more efficient drug delivery system.

## Figures and Tables

**Table 1:** The absorption maxima positions (λ, nm), peak intensities (A), and full widths at half maximum (FWHM, nm) pre and post-centrifugation (CF).

		DOX I		DOX II		DOX III		DOX IV	
Sample	Parameter	Pre CF	Post CF	Pre CF	Post CF	Pre CF	Post CF	Pre CF	Post CF
DOX 2.5 μg/mL	λ (nm)	473.6	472.6	506.7	506.8	533.7	533.6		/
	Intensity (A)	0.042	0.038	0.023	0.022	0.017	0.015		/
	FWHM (nm)	30	30	16	16	11	11		/

ND/DOX [μg/mL/μg/mL] 10/2.5	λ (nm)	474.7	473.16	506.1	506.8	532.6	532.7	575.1	/
	Intensity (A)	0.036	0.033	0.021	0.019	0.016	0.013	0.001	/
	FWHM (nm)	30	30	16	16	11	11	22	/

ND/DOX [μg/mL/μg/mL] 50/2.5	λ (nm)	474.3	475.6	504.5	507.3	534.1	534.1	577.6	579.8
	Intensity (A)	0.023	0.024	0.016	0.013	0.016	0.012	0.004	0.002
	FWHM (nm)	30.01	31	16	16.32	15.4	11.54	18	23.86

ND/DOX [μg/mL/μg/mL] 100/2.5	λ (nm)	477.9	474.5	504.3	506.1	534.4	533.6	574.8	578.8
	Intensity (A)	0.014	0.015	0.013	0.012	0.017	0.011	0.011	0.002
	FWHM (nm)	30	26	16	16	13	12	21	24

ND/DOX [μg/mL/μg/mL] 150/2.5	λ (nm)	479.3	487.7	506.3	511.5	536.1	534.9	575.9	577.3
	Intensity (A)	0.008	0.011	0.015	0.006	0.021	0.009	0.016	0.006
	FWHM (nm)	25	23	16	16	12	12	23	23

**Table 2:** ND-mediated DOX removal ND/DOX binding fractions.

ND Concentration (μg/mL)	A_500_ Post CF	ΔA_500_	DOX Post CF (µg/mL)	DOX Removed (µg/mL/%)	A_580_ Post CF	ΔA_580_	Labile ND/DOX (%)	Stable ND/DOX (%)
10	0.042	0.005	2.4	0.1/4	0.004	0	0	100
50	0.035	0.01	2.05	0.45/18	0.007	0.008	53	47
100	0.028	0.01	1.7	0.8/32	0.008	0.012	60	40
150	0.021	0.014	1.35	1.15/46	0.012	0.014	54	46

Note: initial DOX concentration in all samples was 2.5 µg/mL. Post-centrifugation DOX concentrations were estimated from A_500_ (2.5 µg/mL DOX corresponds to A_500_ = 0.05) and corrected by 0.3 µg/mL to account for CF-associated DOX loss, assuming that free DOX experiences the same procedural loss in the presence of ND. All calculations are based on post-centrifugation absorbance. ΔA_580_ = A_580 pre-CF_ − A_580 post-CF_. Absorbance values of ND/DOX were corrected for the ND background.

**Table 3:** The preview of average sizes with SD value.

ND Size Average				ND/DOX Size Average			
10 μg/mL	50 μg/mL	100 μg/mL	150 μg/mL	10/2.5 μg/mL/μg/mL	50/2.5 μg/mL/μg/mL	100/2.5 μg/mL/μg/mL	150/2.5 μg/mL/μg/mL
96.24	57.04	46.59	52.32	88.10	56.87	56.10	48.89
79.24	59.03	46.16	50.08	96.42	63.26	50.85	47.66
92.16	60.78	45.83	48.77	103.10	65.64	52.53	46.28
SD				SD			
8.87	1.87	0.38	1.80	7.51	4.54	2.68	1.31
After 24 h							
**ND Size Average**				**ND/DOX Size Average**			
10 μg/mL	50 μg/mL	100 μg/mL	150 μg/mL	10/2.5 μg/mL/μg/mL	50/2.5 μg/mL/μg/mL	100/2.5 μg/mL/μg/mL	150/2.5 μg/mL/μg/mL
105.10	78.09	61.39	45.07	107.40	55.42	54.56	47.88
103.90	77.77	61.94	45.26	109.40	54.47	52.62	49.14
106.30	76.97	58.69	45.01	111.60	51.77	51.79	48.81
SD				SD			
1.20	0.58	1.74	0.13	2.10	1.89	1.42	0.65

**Table 4:** Representative effects of ND, DOX, and ND/DOX on cell viability after 48 h.

				The Most Efficient
Cell Line	DOX (2.5 µg/mL)	ND (50 µg/mL)	ND/DOX [50 µg/mL/2.5 µg/mL]	ND/DOX [µg/mL/µg/mL]
U251	54%	82%	52%	50/7.5 (42%)
HS294T	66%	94%	53%	50/7.5 (37%)
MCF-7	66%	88%	68%	50/5 (57%)
MRC-5	72%	82%	69%	100/5 (56%)

**Table 5:** Bliss Independence model values as a practical quantitative approximation of synergy in HS294T cell line.

Cell Line Treatment	ND μg/mL	DOX μg/mL	E_ND_ *	E_DOX_ *	E_expectedND/DOX_ **	E_measuredND/DOX_ *	ΔBliss	Interpretation [42,43]
HS294T 48 h	50	1	0.942	0.807	0.760	0.793	−0.033	Slight antagonism
	50	2.5	0.942	0.658	0.620	0.533	0.087	Synergy
	50	5	0.942	0.560	0.528	0.513	0.015	Mild synergy
	50	7.5	0.942	0.472	0.445	0.373	0.072	Synergy
	100	5	0.858	0.560	0.480	0.507	−0.027	Slight antagonism

* E_ND_, E_DOX_ and E_measuredND/DOX_ are the relative cell viabilities for ND, DOX, and ND/DOX, respectively. ** E_expectedND/DOX_ = E_ND_ ∙ E_DOX_, ΔBliss = E_expectedND/DOX_ − E_measuredND/DOX._

## Data Availability

The datasets generated and/or analyzed during the current study are available from the corresponding author upon reasonable request.

## Abbreviations

The following abbreviations are used in this manuscript:

DOX	Doxorubicin
ND	Nanodiamonds
ND/DOX	Nanodiamonds/Doxorubicin
DMSO	Dimethyl sulfoxide
UV–VIS	Ultraviolet–Visible
DLS	Dynamic Light Scattering
DMEM	Dulbecco’s Modified Eagle’s Medium
FCS	Fetal Calf Serum
EDTA	Ethylenediaminetetraacetic acid
MTT	3-(4,5-dimethylthiazolyl-2)-2,5-diphenyltetrazolium bromide
